# Autophagy in Cancer Stem Cells: A Potential Link Between Chemoresistance, Recurrence, and Metastasis

**DOI:** 10.1089/biores.2014.0035

**Published:** 2015-01-01

**Authors:** Rani Ojha, Shalmoli Bhattacharyya, Shrawan K. Singh

**Affiliations:** ^1^ Department of Urology, Post Graduate Institute of Medical Education and Research, India.; ^2^ Department of Biophysics, Post Graduate Institute of Medical Education and Research, India.

**Keywords:** cancer, cancer stem cells, autophagy, apoptosis, resistance

## Abstract

Cancer cells require an uninterrupted nutritional supply for maintaining their proliferative needs and this high demand in concurrence with inadequate supply of blood and nutrition induces stress in these cells. These cells utilize various strategies like high glycolytic flux, redox signaling, and modulation of autophagy to avoid cell death and overcome nutritional deficiency. Autophagy allows the cell to generate ATP and other essential biochemical building blocks necessary under such adverse conditions. It is emerging as a decisive process in the development and progression of pathophysiological conditions that are associated with increased cancer risk. However, the precise role of autophagy in tumorigenesis is still debatable. Autophagy is a novel cytoprotective process to augment tumor cell survival under nutrient or growth factor starvation, metabolic stress, and hypoxia. The tumor hypoxic environment may provide site for the enrichment/expansion of the cancer stem cells (CSCs) and successive rapid tumor progression. CSCs are characteristically resistant to conventional anticancer therapy, which may contribute to treatment failure and tumor relapse. CSCs have the potential to regenerate for an indefinite period, which can impel tumor metastatic invasion. From last decade, preclinical research has focused on the diversity in CSC content within tumors that could affect their chemo- or radio-sensitivity by impeding with mechanisms of DNA repair and cell cycle progression. The aim of this review is predominantly directed on the recent developments in the CSCs during cancer treatment, role of autophagy in maintenance of CSC populations and their implications in the development of promising new cancer treatment options in future.

## Introduction

Over the decades, we have made a massive leap in our perception of molecular mechanisms involved in tumor formation and its metastatic progression. However, this observation has not directly translated into more effective treatment and cure for patients suffering from cancer. The reasons for the failure of presently available anticancer treatment modalities are related to cellular heterogeneity of tumors. Currently used anticancer drugs target only those cells in the bulk populations that are actively dividing.^1^ Another reason for the inadequacy of cancer treatment is the inherent or therapy-induced resistance of tumor cells to the therapeutic agent.^2^ The inability of conventional treatments to completely eradicate all infiltrative tumor cells is believed to be the major cause of treatment failure as well as recurrence or relapse of tumor. It has been proposed that small subsets of cancer cells, called cancer stem cells (CSCs) are responsible for cancer genesis, growth of tumor, recurrence, and drug resistance of several tumors. CSCs have been identified as immortal tumor-initiating cells that can self-renew and have pluripotent capacity.^3^ To date, CSCs have been discovered in a various solid tumors such as lung,^4^ colon,^5^ prostate,^6^ ovarian,^7^ brain,^8^ and melanoma cancers.^9^ Both CSCs and normal stem cells possess self-renewal capacity; however, the self-regeneration capacity is deregulated in CSCs.^10,11^ It has been hypothesized that traditional cancer therapies reduce the bulk tumor mass but often fail to prevent tumor recurrence and complete remissions. The reason for failure of chemotherapy is due to incomplete eradication of the CSCs population. CSCs therefore represent a potential target for improvement of therapeutic interventions. Significantly, various evidences have reinforced the foundation for emergence of CSC-targeted therapeutic strategies that may help to enhance the efficacy of conventional anticancer therapies.^12^

Solid tumor consists of highly proliferating tumor cells, which are characterized by hypoxic areas arising from an inequity between supply and consumption of oxygen.^13^ The specific hypoxic microenvironments tightly regulate the inherent properties of CSCs. Among the possible mechanisms that have been strongly implicated in the survival of cancer cells in their stressed microenvironment is autophagy.^14^ Various recent groups have focused on the involvement of autophagy in CSCs population.^15–17^ In this review we highlight the topical improvements in our knowledge of autophagy as a drug resistance mechanism in cancer and CSCs as well as the recent strategies to target autophagy as a potential mechanism for augmenting the efficacy of anticancer therapies. Ongoing clinical trials for various cancers involving inhibition of autophagy have also been highlighted, with a view to enlighten the potential for clinical translation in field of cancer.

## Cancer Stem Cells in Cancer

Cancer is a disease caused by the genetic alterations that lead to aberrant gene expression. The aberrant gene expression that results in loss of cell cycle control leads to the increased potential of cancer cell proliferation.^13^ In the process of transformation from normal state to cancerous state, these cells acquire some specific characters/properties called “hallmarks of cancer” These hallmarks include sustained proliferative signals, evading growth suppressor mechanisms, resistance to cell death, indefinite replicative ability, neoangiogenesis, invasion/metastasis, metabolic reprogramming, and evasion of the immune system.^13^ Some researchers also propose loss of differentiation as a separate and important hallmark, because loss of differentiation is the primary difference between benign and malignant tumors.^13^ The cell undergoes a number of pathophysiological changes such as self-sufficiency in growth signals, insensitivity to growth inhibitory signals, evasion of cell death, limitless replicative potential, development of neoangiogenesis, and ability to invade during its transition from normal to cancerous phenotype. These changes are at the metabolic level as well as at the process level. At the metabolic level, there is change in the glucose metabolism and glutamine addiction which are collectively referred as “metabolic reprogramming” of the cancer cells. The importance of metabolic reprogramming is highlighted by the fact that it is now regarded as a separate hallmark of cancer cells. Warburg first reported that cancer cells metabolize glucose to lactate in aerobic condition.^18^

Tumors are a heterogeneous population similar to organs, with multiple cell types that interact with each other and with the extracellular matrix. So it is not necessary for all tumor cells to contain these hallmarks.^13^ Many recent studies propound the CSC hypothesis that suggests the existence of small subsets of neoplastic cells within tumors having an elevated ability to seed new tumors upon experimental implantation in appropriate animal hosts. The existence of CSCs is still an object of skepticism and intense debate but accumulating evidence suggests that CSCs are competent for self-regeneration and differentiation into different cell types. These CSCs were identified and separated based on cell surface markers and characterized by *in vitro* sphere forming ability and tumorigenic potential in various immunocompromised mice models.^19^ The concept of CSC has attractive prospective for identification of CSC-targeted therapies and it is important to determine the crucial molecules regulating the unique properties of CSCs.

## Concept Origin and Hierarchy of Cancer Stem Cells

From more than 150 years ago cancer has been proposed to be initiated from stem cells and this idea reappeared first during 19th century for leukemias^20^ and later for various solid tumors.^21^ It has been reported that cancer consists of phenotypically heterogeneous cells including stromal cells and vasculature. The stochastic model of cancer development proposes that all cancer cells have the ability to give rise to new tumors. However, the CSC hypothesis accentuates that only a small subsets of cancer cells have the potential to generate new tumours containing heterogeneous population of cancer cells. Pierce and Wallace in 1971 showed that undifferentiated malignant cells give rise to benign well-differentiated cells, which indicates the presence of cellular heterogeneity or hierarchy of tumors cells.^22^ Based on these studies, it was believed that CSCs with deregulated self-regeneration and differentiation were responsible for tumor initiation and progression. The existence of CSCs is being confirmed in number of different tumor types such as leukemias,^23,24^ urothelial carcinoma,^25^ breast carcinoma,^26^ colon carcinoma,^27^ head and neck carcinoma,^28^ ovarian carcinoma,^29^ pancreatic carcinoma,^30^ liver cancer ^31^ and ewing sarcoma.^32^ CSCs are undifferentiated cells with self-renewal ability and can differentiate into multiple lineages. Once CSCs forced to differentiate, these cells lose their quiescent properties and be converted into more sensitive to chemotherapy.^33^ Although CSCs have shown to contain rare subsets of cancer cells, rarity in terms of tiny percentage is not a key measure, and is not essentially a defining characteristic in all cancers.^12^ Collectively, a new paradigm has been established that the ability to initiate tumors and to give rise to the heterogeneous cell populations found in the original tumor is exclusively attributed to the CSC population with all of their differentiated progeny lacking these features.^34^ A recent study by Auffinger et al. has provided experimental evidence that glioma cells exposed to chemotherapeutic agent temozolomide were able to interconvert between nonglioma stem cells and glioma stem cells, thereby replenishing the original tumor population. This led to a more infiltrative phenotype, resulting in enhanced chemoresistance.^35^ This may represent a potential mechanism for tumor relapse. Therefore, understanding the mechanisms underlying the maintenance of CSCs is vital for the development of new therapeutic strategies that might be capable to target the specific population of CSCs.

## Therapeutic Potential of Cancer Stem Cells

**FIG. 1.**
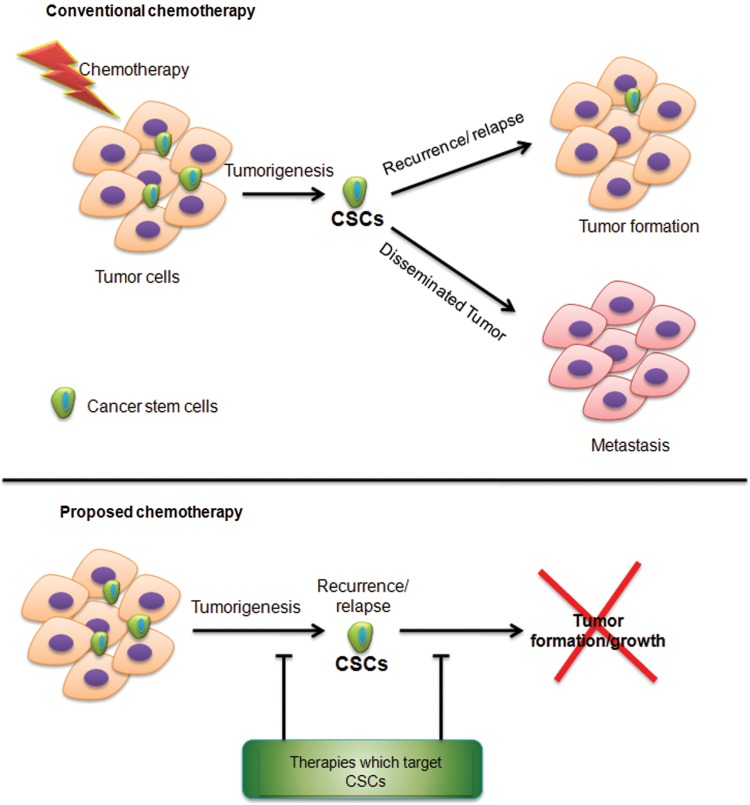
Conventional and proposed chemotherapeutic strategies in cancer treatment: Tumor cells are heterogeneous and include cancer stem cell (CSC) populations. Chemotherapy can reduce tumor burden by eliminating the highly proliferative cells, and relatively dormant cells or CSCs are spared. These chemotherapy-resistant cells can seed a new cancer by promoting tumor growth and metastasis. Thus, chemotherapy that also targets CSCs might be beneficial for preventing or inhibiting tumor regrowth or recurrence.

A close relationship exists between CSCs, tumorigenesis, drug resistance, and invasion; therefore, seclusion of these cells is a requisite for targeting them. Various specific surface biomarkers have been identified to differentiate CSCs from bulk tumor cells as well as the normal stem cells. Presently, fluorescence-activated cell sorting is the most common method to identify CSCs. This method is based on identification of specific cell surface markers or intracellular molecules.^36^ However, the CSC markers are not very reliable, since CSCs may not all express the markers. On the other hand, some non-CSCs may also express them. Thus, the markers might not be able to unambiguously isolate all of the CSCs but these can be used to identify the CSC-rich subpopulations.^36^ The CSCs rely on their microenvironment, which make targeting CSCs within a cancer mass a intimidating task. Still, CSCs populations might be more relevant in the ultimate cancer prognosis. Thus, a better understanding of the molecular signaling underlying CSC pathology will help in designing new therapeutic targets and novel strategies for the successful treatment of cancer (Fig. 1).

Conventional anticancer therapeutic approaches are directed primarily at bulk tumor cell populations. Such strategies have inadequate efficacy because of inherent or acquired drug resistance.^37^ CSCs chemoresistance has been reported in human leukemias, melanoma, brain, breast, pancreatic, and colorectal cancers.^38^ Recently, numerous groups have started clinical trial on patients with lung, pancreatic, brain, and breast cancer and used drugs that can target pathways involved in CSCs development.^39,40^ The fate of stem cells is determined by stem cell niche, which comprises of stromal cells, cytokines, and growth factors.^41–44^ Recently, it has been demonstrated that unfavourable niches may drive good stem cells into bad ones leading to generation of CSCs.^45–47^ If CSCs are the main perpetrator of tumor development or relapse and cause of therapeutic resistance, treatment approaches that target CSCs could potentially improve the efficacy of presently available treatment regimens. Recently, autophagy has been shown to help in acquisition of resistance in CSCs towards anticancer therapy in various cancers.^48^ However, the therapeutic promise of autophagy modulation in CSC is yet to be verified experimentally.

## Role of Autophagy in Cancer

**FIG. 2.**
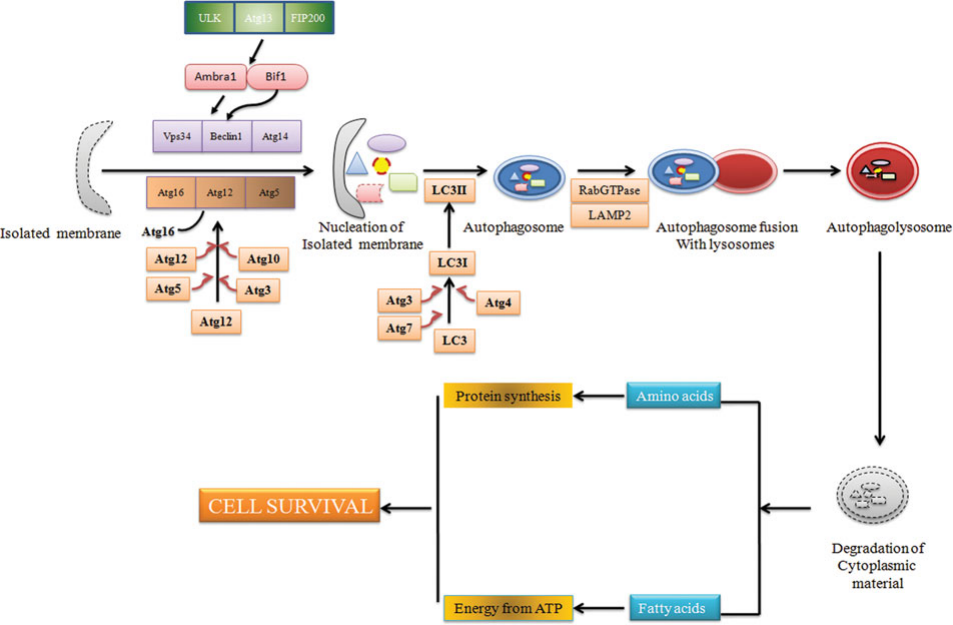
Schematic diagram of biogenesis of an autophagosome. Autophagy involves the degradation of cytosolic proteins and organelles in the lysosomes via double-membraned structures called autophagosomes which are formed from pre-autophagosomal structures (PASs) or isolated membrane. The membrane source involved in autophagosome biogenesis may involve contributions from endoplasmic reticulum, mitochondria, and plasma membrane. Atg13-ULK1 and Beclin-Vps34 complexes regulate the initiation of PAS formation. Two ubiquitin-like conjugation systems [Atg5–Atg12 conjugation and LC3–phosphatidyl ethanolamine (PE) conjugation] are involved in the elongation of PAS. The Atg5–Atg12 conjugation involves Atg7 (E1-like ubiquitin ligase) and Atg10 (E2-like ubiquitin ligase), while Atg7 and Atg3 act as the E1-like and E2-like, respectively, in LC3-PE conjugation. The Atg12–Atg5 is noncovalently conjugated to Atg16L1 (Atg12–Atg5.Atg16L1), resulting in an 800-kDa complex containing Atg12–Atg5. The Atg12–Atg5.Atg16L1 complex exhibits an E3-like ubiquitin ligase activity toward LC3–PE conjugation. Rab-GTPase and LAMP2 complex is involved in the fusion step of autophagy. After formation of autophagolysosomes, cytoplasmic material is degraded and transported to the cytosol wherein degraded biomolecules are used for the maintenance of cellular homeostasis.

Autophagy is referred as a highly regulated conserved catabolic process that functions as a cell survival mechanism during cellular stresses like starvation, hypoxia, and chemo/radiotherapy.^49^ Autophagosomes that engulf damaged organelles or particles are formed due to activation of autophagy. Eventually, these autophagosomes fuse with lysosome to form autophagolysosomes. Lytic enzymes within the autophagolysosomes degrade its interiors to provide cells the nutrients such as amino acids or fatty acids necessary for cell metabolism (Fig. 2). Defects in the autophagy machinery has been shown to be associated with neurodegeneration, and muscular dystrophy, as well as a variety of cancers.^50^

Autophagy has been reported to have a dual role in cancer. It acts as a tumor suppressor by preventing the accumulation of damaged proteins/organelles. In some cases, it plays the role of tumor enhancer by maintaining cellular homeostasis under nutrient deprivation and hypoxia. Tumor cells activate autophagy in response to cellular stress or increased metabolic demands of cancer cells.^51^ Autophagy-mediated stress tolerance can facilitate cell survival by sustaining energy production that can lead to tumor growth and therapeutic resistance.^52^ Preclinical studies have shown that autophagy inhibition restored chemosensitivity and increased the tumor cell death in various cancer. These results consolidated autophagy as a therapeutic target. This led to multiple clinical trials in humans to evaluate the potential role of autophagy inhibition using hydroxylchloroquine (HCQ) in combination with chemotherapy or targeted agents. The role of autophagy and its regulation in cancer cells continues to come out, and further research aims to delineate optimal strategies to modulate autophagy for therapeutic improvement.^53^

### Autophagy as a tumor suppressor

The tumor suppressor properties of Beclin1 were first identified by assessing the tumorigenicity in immuno-compromised mice. It was observed that transfection of Beclin1 in the breast cancer cell line MCF7 decreased the proliferation rate and reduced the malignant phenotype.^54^ Cells treated with an estrogen antagonist, tamoxifen, caused cell death with typical autophagic characteristics.^54^ In another study, cells treated with combination of estradiol and 3-MA (autophagy inhibitor) inhibited the cell death.^55^ Similarly, treatment of arsenic trioxide induced G2/M arrest and autophagic cell death in malignant glioma cell lines.^56^ Radiation treatment has been shown to induce autophagic cell death in cell lines from breast, prostate, colon cancer and glioblastoma multiforme by decreasing cell proliferation and increasing the autophagic activity.^57,58^ Recently, Han et al. showed that sulfasalazine, an anti-inflammatory drug, promotes autophagic cell death via protein kinase B (Akt) and extracellular signal regulated kinase (ERK) pathways and has chemotherapeutic potential for the treatment of oral cancer.^59^ In line with this, Lu et al. also showed that treatment of cyclovirobuxine-D induces autophagy-associated cell death via the Akt/mammalian target of rapamycin (mTOR) Pathway in MCF-7 cells.^60^ Likewise, Aryl et al. demonstrated that the anticancer effects of baicalein, a flavonoid, are mainly due to autophagic cell death through activation of the AMP-Associated protein kinase (AMPK)/Unc-51 like autophagy activating kinase 1 (ULK1) pathway and inhibition of mTOR/Raptor complex 1 expression.^61^ These results provide new mechanistic insights into the anticancer functions of autophagy inducers which may be used as potential therapeutics for cancer treatment.

### Autophagy as a tumor enhancer

The major function of autophagy reported in cancer cells is to provide tolerance during stress to maintain tumor cell survival. Autophagy is a mechanism to maintain cellular integrity during metabolic stress, drug treatment or radiation damage.^62–65^ Accordance with this, in the absence of autophagy, DNA damage, gene amplification, and chromosomal abnormalities were evident during metabolic stress in various cancer cells.^62,63^ Inhibition of autophagy has been shown to enhance the death response to radiotherapy in breast, prostate, colon, and in malignant glioma cells.^64,65^ Similarly, inhibition of autophagy increased the anticancer potential of the histone deacetylase inhibitor, suberoylanilide hydroxamic acid (SAHA) in imatinib-resistant primary chronic myeloid leukemia (CML) cells,^66^ and the anti-angiogenic effects of kringle 5 in endothelial cells, by initiating apoptotic cell death.^67^ Recently, Tran et al. showed that autophagy inhibitor 3-methyladenine augments the apoptotic effect of tocotrienols in breast cancer cells.^68^ Similarly, two recent reports by Ishaq et al.^69^ and Ojha et al.^70^ also reported that inhibition of autophagy potentiates cytotoxic effect of anticancer agents in bladder cancer cell lines and primary bladder cancer cells. Another recent report also showed that inhibition of autophagy by chloroquine sensitizes HT-29 colorectal cancer cells to concurrent chemoradiation.^71^

These examples represent a general mechanism for context-specific regulation of cell fate by autophagy. Thus, it may be suggested that autophagy can regulate cancer cell death both positively and negatively.

## Autophagy Modulation in Tumor: An Emerging Concept in Cancer Therapy

Certain therapeutic approaches to cancer, including radiation and cytotoxic drugs that have been known to activate apoptosis, also induce autophagy in different cancer cell lines. Chloroquine (CQ) and HCQ, autophagy inhibitors which prevent autophagosomal maturation, have been shown to increase the anticancer activity of various chemotherapeutic drugs in different cancer cells. Administration of bortezomib with HCQ is in clinical trial in refractory multiple myeloma. HCQ and ixabepilone have shown a therapeutic improvement in breast cancers, and the combination of HCQ, radiation and temozolomide are in clinical trials in patients with glioblastomas. In CML, cell death was observed by the combined treatment of CQ and the histone deacetylase inhibitor suberoylanilide hydroxamic acid (SAHA).^72,73^ Altogether, it appears that addition of CQ or HCQ can inhibit autophagy-dependent survival and augment their anticancer activity.

However, rising evidences reveal that the ability of CQ and its derivative to impede autophagy may not be the sole process by which they exhibit anticancer activity. CQ and HCQ may also involve other pathways such as lysosomal membrane permeabilization which can help to induce antitumor effect on cancer cells.^74^ Hence, advance knowledge of the cellular targets and signaling network of CQ or HCQ should be kept in mind for the ongoing clinical trials where CQ or HCQ are used as autophagy inhibitors.

The anticancer drugs reported to induce autophagy include imatinib, a BCR-ABL tyrosine kinase inhibitor;^75^ cetuximab, an anti-epidermal growth factor receptor;^76^ proteosome inhibitors;^77^ tumor necrosis factor-related apoptosis-inducing ligand;^78^ and vorinostat and OSU-HDAC42, histone deacetylase inhibitors.^79^ Furthermore, agents like tamoxifen, cyclooxygenase inhibitors, and the protease inhibitor nelfinavir, reported to have diverse mechanisms of action, have also been shown to induce autophagy in various tumor cells.^80^ Given that various cancer cells undergo autophagy after anticancer therapies, we propose to use autophagy inhibitor to our benefit to kill cancer cells. Inhibition of autophagy may induce apoptosis, thus resulting in a significant cell death than is achievable with currently available anticancer therapies. By modulating the autophagic pathways, we might be capable to design more effective anticancer strategies. However, further studies will be needed to clarify how to manipulate autophagic pathways before such new therapies can be developed. A list of various clinical trials based on autophagy modulation has been list in Table 1.

**Table 1.** **Examples of Clinical Trials Combining the Autophagy Inhibitor Hydroxylchloroquine as an Adjunct to Anticancer Therapies**

Tumor	Interventions	Clinical trial number
Multiple myeloma	HCQ+bortezomib	NCT00568880
Brain, central nervous system tumors	HCQ+temozolomide/radiation therapy	NCT00486603
Prostate cancer	HCQ+docetaxel	NCT00786682
Prostate cancer	HCQ (after local therapy)	NCT00726596
Breast cancer	HCQ+ixabepilone	NCT00765765
Breast cancer	HCQ	NCT01292408
Ductal carcinoma *in situ*	CQ+tamoxifen	NCT01023477
Lung cancer	HCQ+bevacizumab/ carboplatin paclitaxel	NCT00728845
Pancreas cancer	HCQ+gemcitabine	NCT01128296
Pancreatic cancer	HCQ+gemcitabine/abraxane	NCT01506973
Pancreatic cancer	HCQ+capecitabine+photon radiation	NCT01494155
Renal cancer	HCQ (patients with resectable renal cell carcinoma)	NCT01144169
Renal cell carcinoma	HCQ+interleukin-2	NCT0155036
Renal cell carcinoma	HCQ and RAD001	NCT01510119
Advanced solid tumors or prostate or renal cancer	HCQ+Akt inhibitor/ MK2206 (MK-2206)	NCT01480154
Adult solid tumors	HCQ+temozolomide	NCT00714181
Adult solid tumors	HCQ+temsirolimus	NCT00909831
Adult solid tumors	HCQ+vorinostat	NCT01023737
Advanced cancer	HCQ+sunitinib	NCT00813423
Metastatic solid tumors	HCQ+temsirolimus	NCT00909831
Advanced solid tumors	HCQ+vorinostat	NCT01023737
Colorectal cancer	HCQ+XELOX+bevacizumab	NCT01006369
Colorectal cancer	HCQ+FOLFOX/ bevacizumab	NCT01206530
Metastatic colorectal cancer	HCQ+capecitabine, oxaliplatin, and bevacizumab	NCT01006369
Melanoma	HCQ (after surgery)	NCT00962845
Solid tumors undergoing radiation therapy for bone metastases	HCQ	NCT01417403
NSCLC	CQ+cisplatin/ etoposide	NCT00969306
NSCLC	HCQ+gefitinib	NCT00809237
NSCLC	HCQ+paclitaxel and carboplatin	NCT01649947
Advanced or recurrent NSCLC	HCQ+carboplatin, paclitaxel, bevacizuma	NCT00933803
Advanced NSCLC and (EGFR) mutations	HCQ+erlotinib	NCT00977470
Chronic myeloid leukemia	HCQ+imatinib	NCT01227135

CQ, chloroquine; EGFR, epidermal growth factor receptor; FOLFOX, 5-Flurouracil, leucovorin, and oxaliplatin; HCQ, hydroxylchloroquine; NSCLC, non-small cell lung cancer; XELOX, capecitabine plus oxaliplatin.

Autophagy has also been shown to either precede or act in parallel with another cell death mechanism called apoptosis.^81–83^ Autophagic cell death is induced in leukemia and glioma cells via regulation of the mitochondrial stress sensor BNIP3 malignant glioma by arsenic trioxide.^84^ It has been demonstrated that autophagy precedes caspase-dependent apoptosis.^69^ Therefore, the induction of autophagy may exert other promises, which should be considered during designing of new treatments for these malignancies. However, the consequences of promoting autophagy in tumor cells are partly understood and may depend on multiple factors, including the extent of induction, duration, cellular context, and cell types.

Solid tumors usually grow in low oxygen environments and are associated with an increased angiogenesis, which makes them more aggressive, with higher invasive capacity. Though advances have been made in understanding the role of hypoxia in the stem cell niche, very less is known about the potential role of hypoxia in maintaining the cancer stem cell niche. Hypoxia-inducible factor (HIF), a master transcriptional factor in nutrient stress signaling, has been shown to regulate intracellular pH, metabolism, cell invasion and autophagy.^85^ Taken together, it appears that tumor microenvironment regulate autophagy as well as CSCs niche. Therefore, targeting autophagy in CSCs may aid to improve tumor recurrence or metastasis.

## Autophagy in Cancer Stem Cells

CSCs are believed to dependent on their own microenvironment to sustain the population. Recently, autophagy has been shown to a major factor for CSCs survival and resistance.^16,17^ In addition, autophagy has been reported to play an important role in the maintenance dynamic equilibrium between CSCs and normal stem cells.^86^ In the sections that follow we shall discuss the involvement of autophagy in CSCs in various cancers.

### Colon cancer stem cells

Kantara et al. had shown that curcumin led to the survival of colon CSCs. At optimal concentrations, curcumin greatly reduced expression levels of stem cell markers. Unexpectedly, curcumin increased proliferation and autophagic survival of CSCs. Spheroid cultures were disintegrated by curcumin *in vitro* but regrew within 30 to 40 days of cisplatin treatment. This finding proposes the survival benefit from autophagy, permitting long-term persistence of colorectal cancer.^87^

### Breast cancer stem cells

Sanchez et al. demonstrated that serum-deprived mesenchymal stem cells (SD-MSCs) supported MCF-7 tumor growth. SD-MSCs-injected tumors exhibited higher cellularity, decreased apoptosis, and differentiation. Beclin1 staining indicated autophagic areas surrounded by actively proliferating cells. In addition, *in vitro* studies demonstrated that SD-MSCs survive using autophagy and secrete paracrine factors that support tumor cells following nutrient/serum deprivation.^88^

Another study by Chatterjee et al. showed that autophagy markers like Atg5, Atg12, and LC3B were overexpressed in dormant stem cell–like breast cancer cells. Inhibition of autophagy by 3-methyladenine reversed the dormant phenotype. In addition, these authors demonstrated that the c-jun NH2 terminal kinase (JNK/SAPK) was unregulated in dormant stem cell–like breast cancer cells and were responsible for increasing autophagy.^89^

Gong et al. showed that expression of Beclin1 (an autophagy protein) was increased in mammospheres derived from human breast cancers. Similar findings were observed in other breast cancer cell lines (MCF-7, BT474). The level of basal and starvation-induced autophagy flux was found to be higher in aldehyde dehydrogenase1-positive population. The authors clearly demonstrated that Beclin1 was crucial for maintenance of CSC and tumor development in athymic mice. This study highlighted role of the autophagic pathway for CSC maintenance.^90^ Collectively, these findings signify that CSCs utilize autophagy for tumor survival and growth.

### Pancreatic cancer stem cells

The role of HIF-1α and autophagy in modulating conversion of non-stem pancreatic cancer cells to stem cells was studied by Zhu et al. in 2013.^86^ These authors reported that higher autophagic flux was associated with the increased expression of HIF-1α. They suggested a specific role of HIF-1α and autophagy in promoting the dynamic equilibrium between CSCs and non-CSCs.^86^ This study emphasized the importance of developing therapeutic strategies targeting CSCs as well as the microenvironmental influence on the tumor.

### Chronic myeloid leukemic stem cells

Bellodi et al. showed that suppression of autophagy related genes increased cell death induced by imatinib mesylate (IM) in cell lines and primary CML cells.^16^ Combination of autophagy inhibitor with tyrosine kinase inhibitors (TKI) like IM, nilotinib, or dasatinib, resulted in complete elimination of phenotypically and functionally defined CML stem cells.^16^ This finding suggested that autophagy inhibitors may enhance the therapeutic effects of TKIs in the treatment of CML.

### Urinary bladder cancer stem cells

Recently, Ojha et al. reported that side population (SP), a subset of CSCs of urinary bladder cancer cells (T24 and UM-UC-3) possessed higher mRNA expression of stemness genes. These cells also showed greater tendency to form spheroid in nonadherent conditions as compared with other bulk cells or NSP.^48^ The SP cells showed substantial resistance to gemcitabine, mitomycin and cisplatin treatment compared with the NSP counterpart. A high autophagic flux in SP cells was associated with resistance of SP cells to chemotherapy. Both pharmacological and small interfering RNA–mediated inhibition of autophagy potentiated the chemotherapeutic effects of gemcitabine, mitomycin and cisplatin in these cells.^48^ Thus, autophagy is associated with cell survival in bladder carcinoma and may be a potent target for developing more effective treatment to enhance patient survival.

### Brain tumor stem cells

Contrary to the above findings about the role of autophagy in cancer stem cells, Jiang et al. (2007) showed that Delta-24-RGD, anti-glioma agent, induced cell death by accumulation of autophagic proteins and autophagic vacuoles in brain tumor stem cell lines derived from surgical glioblastoma specimens. These samples were also found to express high levels of adenoviral receptors.^17^ Treatment of Delta-24-RGD in brain tumor stem cells derived from xenografts showed significantly improved the survival of glioma-bearing mice. Immuno-fluorescence analysis showed high levels of Atg5 expression which indicated that Atg5 might be useful a surrogate marker of the anti-glioma effect. In conclusion, they showed that brain tumor stem cells were susceptible to adenovirus-mediated cell death via autophagy both *in vitro* and *in vivo*. This study signified that the brain tumor stem cells were the cause of sustaining tumor growth and hence developing therapies to target the brain tumor stem cells might be a more effective strategy than conventional therapy.

Based on the existing studies, we hypothesized that the combination of autophagy modulators with chemotherapeutic agents may emerge as potentially effective anticancer therapies. However, there are many bottlenecks before developing a successful anticancer therapy based on autophagy modulation, as autophagy response vary according to cell type, stress, and stimulus. The impact of the tumor microenvironment on autophagy function needs to be demonstrated experimentally. Also, new and reliable methods for quantifying autophagy in clinical samples need to be developed. Apart from this, understanding the role of autophagy in the regulation of therapeutic sensitivity can overcome chemotherapy resistance and sensitize the tumor cells to anticancer therapies are certain factors to be considered before embarking on a therapy.

All together, it appears that the biology of autophagy is still not clear with respect to its functional aspects. However, at this point we can only say that the fate of autophagy may depend on various factors like stimulus, cell type, and microenvironment. Therefore, understanding the molecular mechanism, signaling cascade, and involvement of regulatory pathways involved in autophagy will be important in determining the physiological role of autophagy in cancer stem cells and exploring therapeutic strategies. New and exciting autophagy modulators for more effective and safe anticancer strategies are worthy of further investigation.

## Conclusions

**FIG. 3.**
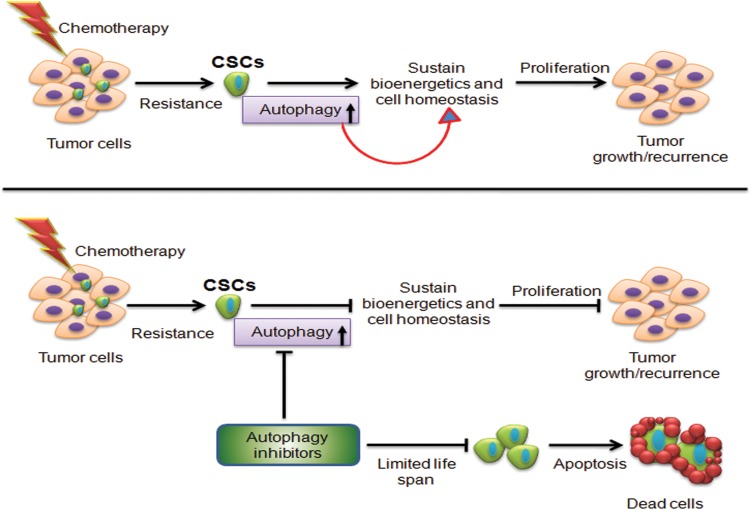
Proposed model for the role of autophagy in cancer stem cells. Tumor consists of a heterogeneous cell population composed of clones from dividing tumor cells and a few tumor initiating cells or cancer stem cells (CSCs). Conventional therapies like radiotherapy and cytotoxic chemotherapy kill the dividing cells, but the tumor-initiating CSCs remain unaffected via autophagy-mediated cell survival mechanism. Therefore, targeting autophagy in CSCs may help to overcome the resistance and relapse of tumor.

For most cancers, survival rates have remained unchanged for decades and systemic disease is almost always fatal. Experimental and clinical data provide an emergent body of evidence supporting the hierarchical organization of cancers with a small number CSCs able to self-renew, repopulate a tumor after treatment, and initiate metastatic growth. The resistance of CSCs to chemotherapy and their relative resistance to radiotherapy enlighten why macroscopic tumor response to anticancer treatments is not a robust predictor for clinical outcome. However, most established chemotherapies continue to be developed based on their effects on bulk tumor cell populations, since it is still not apparent how to utilize the knowledge about CSCs in drug screening. We are still far away from mounting practical tools for screening new drugs and drug combinations that will allow us to eliminate CSCs from bulk tumor. However, it is worth noting that autophagy is a predominant factor that helps in the acquisition of resistance to chemotherapy. A major chunk of preclinical data suggests that stress-induced autophagy in CSCs help in their survival and inhibition of autophagy can overcome CSC resistance. These data establish autophagy as a novel therapeutic target whose modulation presents new opportunities for cancer treatment (Fig. 3).

## Abbreviations Used

AMPK: AMP-Associated protein kinase
Akt: protein kinase B
CQ: chloroquine
CSC: cancer stem cells
EGFR: epidermal growth factor receptor
ERK: extracellular signal regulated kinase
FOLFOX: 5-Flurouracil, leucovorin, and oxaliplatin
HCQ: hydroxylchloroquine
HIF: hypoxia-inducible factor
IM: imatinib mesylate
mTOR: mammalian target of rapamycin
NSCLC: non-small cell lung cancer
PAS: Pre-autophagosomal structure
PE: phosphatidyl ethanolamine
SAHA: suberoylanilide hydroxamic acid
SD-MSC: serum-deprived mesenchymal stem cells
SP: side population
TKI: tyrosine kinase inhibitors
ULK1: Unc-51 like autophagy activating kinase 1
XELOX: capecitabine pluse oxaliplatin
